# Closing the life expectancy gap: An ecological study of the factors associated with smaller regional health inequalities in post-reunification Germany

**DOI:** 10.1016/j.socscimed.2024.117436

**Published:** 2024-10-19

**Authors:** Julija Simpson, Viviana Albani, Andrew Kingston, Clare Bambra

**Affiliations:** a Population Health Sciences Institute, Newcastle University, Ridley 1 Building, Newcastle upon Tyne NE1 4LP, UK; b Population Health Sciences Institute, Newcastle University, Biogerontology Building, Campus for Ageing and Vitality, Newcastle upon Tyne, NE4 5PL, UK

**Keywords:** Health inequalities, Social policy, Life expectancy, Political economy

## Abstract

Regional health inequalities are rising globally. The case of German reunification offers a unique opportunity to explore how such inequalities can be reduced, or even eliminated: following reunification, a long-standing life expectancy gap between East and West Germany was closed for women and markedly reduced for men in less than 15 years.

We used data from official national statistics covering the period 1994–2020 for 15 regions in East and West Germany. Using fixed-effects models with an interaction term for regions in the East, we investigated whether within-region changes in key hypothesised factors (social security expenditure, healthcare improvements, changes in alcohol consumption, and life satisfaction), have had differential impacts on life expectancy at birth and at 65 years.

Our results show that increases in social security benefits in the East following reunification has been the most important factor for lowering inequalities between the two parts of Germany: for every 10% increase in social security benefits, life expectancy at birth increased by an additional 1.05 [0.68; 1.41] months for males and by 0.57 [0.18; 0.97] months for females in East relative to West Germany. We find the protective effect of social security benefits also for women at 65 years (additional 0.38 [0.06; 0.70] months) but not for men.

Our findings suggest that increasing social security expenditure could be an effective policy tool for reducing health inequalities across regions with different levels of economic development. This provides additional support for the materialist hypothesis and the political economy theory of the root causes of health inequalities.

## 1 Introduction

Subnational regional inequalities in mortality are large and increasing within most developed countries. An important factor associated with such disparities is the uneven social and economic development within countries (Keenan et al., 2022). For example, in the UK, regional inequalities in life expectancy have been shown to follow a socio-spatial gradient – the more deprived the region, the lower the life expectancy (Marmot, 2020). Similarly, the longstanding health advantage of the north of Italy is related to its higher economic growth (Bambra, 2016), whereas in the USA, the distribution of economic conditions across the states has been an important determinant of regional variation in women’s mortality (Montez et al., 2016). There is a growing recognition that addressing regional health inequalities is important – not only as a matter of social justice but also because of their importance for economic growth and development (European Commission, 2013; Bambra et al., 2018; Marmot, 2020).

The case of German reunification offers a unique opportunity to explore how such inequalities can be reduced, or even eliminated: following reunification in 1990, the long-standing life expectancy gap between East and West Germany was closed for women and markedly reduced for men in a period of less than 15 years. Factors contributing to this achievement, however, are multifaceted and not yet fully understood (Grigoriev and Pechholdová, 2017).

Providing a better understanding of the changes that enabled Germany to ‘close the life expectancy gap’ in such an epidemiologically short time frame is the key aim of our study. In doing so, we will also contribute to the wider health inequalities literature on the relative contribution of the material, psychosocial and behavioural causes of health inequalities (Skalická et al., 2009; Moor et al., 2017). Identifying the factors leading to falling health inequalities in Germany could also aid the development of regional policies in comparable countries.

### 1.1 Health inequalities in Germany pre- and post-reunification

After the Second World War (between 1949 and 1990), Germany was split into two separate states: the German Democratic Republic (GDR/East Germany) – a Communist totalitarian state, part of the Warsaw pact or Soviet bloc; and the Federal Republic of Germany (FRG/West Germany) – a liberal democratic and capitalist state. This also included – and was symbolised by – the division of Berlin into two halves – East and West – by the Berlin wall.

Following the separation, East Germans inhabited a markedly different world from West Germans (and the citizens of other wealthy democracies). The GDR was a totalitarian regime, dominated by a single political party – the Socialist Unity Party of Germany, whose state communist ideology was enforced across all parts of society. Under this regime, East Germany fell behind the West in terms of key indicators including economic development (e.g., GDP), healthcare provision and medical technology, and living standards (Fulbrook, 2005; Becker et al., 2020)

In terms of population health, between 1949 and 1960, life expectancy was rising at very similar rates in East and West Germany (Becker et al., 2020). Substantial gains in life expectancy during this period were also apparent in other Soviet Bloc countries, such as Hungary (Cockerham, 1997). These improvements were largely attributed to rapid progress in combating infectious disease, facilitated by effective public health policy (Tulchinsky and Varavikova, 1996; Mackenbach, 2013). However, from the 1960s, life expectancy began to stall and, in some cases, to deteriorate in the Soviet Bloc countries. In line with this broader trend (albeit not as extreme), East German life expectancy started to lag behind the West from 1970 – primarily due to a rise in circulatory diseases, which the chronically underfunded and economically inefficient healthcare system was ineffective in addressing (Cockerham, 1997).

The notable lack of progress in improving population health (as well as living standards) was apparent despite the expansion of the welfare state in 1971 (Burdumy, 2013). This is likely because most social security benefits were tightly linked to workplace and therefore oriented towards working-age population, leaving most pensioners in poverty. Their access to healthcare was also limited as the system prioritised people of working age (ibid). Thus, it is perhaps unsurprising that the greatest contributors to the East-West life expectancy gap were the older (60+) age groups (Grigoriev and Pechholdová, 2017)

It is important to note that such marginalisation is not a characteristic of the more typical socio-democratic welfare states, or socialism more generally. While socialism as an ideology aims to promote welfare for all, the GDR was ruled by an externally imposed totalitarian regime, with its citizens lacking basic political freedoms, making it unrepresentative of a truly socialist state (Navarro, 1992: p.585), despite the ideological claims.

Following the collapse of Soviet regime, most post-socialist economies in Eastern Europe experienced a significant economic shock and contraction in welfare provision, both in terms of benefit levels and eligibility (Aidukaite, 2003). The economic shock of transition to a market economy was in turn accompanied by an unprecedented mortality crisis and widening health inequalities (Stuckler et al., 2009; Bessudnov et al., 2012). In contrast, both East German healthcare and social security systems received significant investments from the West following reunification. This transformation was accompanied by rapidly narrowing regional health inequalities, with the life expectancy gap narrowing significantly for men (from 3.5 to 1.5 years between 1990 and 2020) and eliminated for women – a phenomenon that has been described by demographers as a ‘remarkable achievement’ (Max Planc Institute for Demographic Research, 2020).

### 1.2 Closing the gap: plausible mechanisms

To understand the factors behind the narrowing of the life expectancy gap between the more economically deprived East and the more affluent West Germany following reunification, we draw on the political economy of health approach (Bambra et al., 2005, 2019; Bambra, 2016). The political economy approach asserts that the social and behavioural determinants of the health of populations and places are shaped by macro-level structural determinants including politics and policies, the economy, and public institutions and that, even within the constraints of unequal societies, the determinants of health are amenable to public policy interventions (Bambra et al., 2005, 2019; Beckfield et al., 2015; Bambra, 2016).

We use this approach to examine the changes to the material, behavioural and psychosocial determinants of health which occurred because of Germany’s economic and social reunification policies. Below we outline each of these pathways in relation to the literature on their potential role in closing the health gap in post-reunification Germany.

#### 1.2.1 Material pathway

The ‘materialist’ explanation of health inequalities posits that differences in living standards can result in health inequalities due to differences in means to access to goods and services and exposures to physical risk factors (such as poor working conditions) (Skalická et al., 2009; Bartley, 2016). Therefore, reducing disparities in living standards should result in lower health inequalities.

Bringing up material living conditions in the East of Germany in line with those in the West was one of the key aims of reunification. Amongst the main policy tools in achieving this aim were the substantial public transfers to the East, largely consisting of social security and healthcare investments (Enenkel and Rösel, 2023). Within less than ten years post-reunification, pensions in East Germany caught up with those in the West and, for women (given their longer working histories in the East) retirement benefits even started exceeding the average for women in the West – marking a significant expansion in living standards, especially considering that before reunification, Eastern German pensions were less than half (40%) of those in the West (Vogt and Kluge, 2015).

At the same time, considerable support was given to modernise equipment used by private physicians and hospitals in East Germany. Within just a few years of reunification, the eastern medical system reached the standards of that in the West, allowing East Germans to access up-to-date diagnostic and treatment technologies, medical information as well as efficient drugs (Nolte et al., 2002). The improvements were particularly evident in medical technology, as prior to reunification, East Germany suffered from substantial shortages of technical equipment. For example, prior to 1990, there was one ultra-sound device per 32,000 inhabitants in East Germany compared with one per 2500 in the West (Schroeder and Deutz-Schröder, 2008). After reunification, East Germany adopted the western healthcare system and, in turn, their clinical care pathways – increasing the per capita healthcare budget to match Western levels (Vogt and Kluge, 2015; Rehnberg, 2020).

A growing body of evidence suggests that improvements in material living conditions, particularly in healthcare, were important contributors to the narrowing life expectancy gap between East and West Germany. For example, a study by Nolte et al. (2002) has found that improvements in temporary life expectancy (between birth and age 75) in East Germany were largely driven by reductions in conditions amenable to either secondary or primary prevention. Similarly, studies focusing on the elderly population (aged 65 years and over) have also found that improved healthcare availability and technology could have led to improvements in life expectancy for this group (Vogt and Vaupel,2015; Grigoriev and Pechholdová, 2017).

To the best of our knowledge, only one study investigated the effects of increased social security spending. A study by Vogt and Kluge (2015) explored the effects of increasing welfare spending on mortality differentials between East and West Germany during the period before and after reunification (1980–2000). To estimate this, the authors used a linear regression model, with spending measures aggregated by year and sex, and separate models for East and West Germany. The key finding of the study was that every euro invested in public transfers (in East German pensions and healthcare) yielded on average three additional hours in life expectancy. Public spending increases (especially in healthcare) were found to be particularly important for older age groups, with pension spending also being important, albeit to a lesser extent. This is in keeping with a growing body of international evidence, demonstrating the importance of social security for reducing health inequalities (Lundberg et al., 2008; Beckfield and Bambra, 2016; Alexiou et al., 2021; Simpson et al., 2021).

In summary, the evidence on the ‘material pathway’ points to improvements in both healthcare and social security as potential explanations for the reductions in the East-West health gap. However, more evidence – particularly with regards to social security – is still needed to establish their relative contribution. Our paper addresses this gap.

#### 1.2.2 Behavioural pathway

The behaviouralist explanation suggests that health inequalities are a result of adverse health behaviours related to individual choices, or due to greater cultural acceptance of such behaviours in some places (Skalická et al., 2009; Bartley, 2016). Improving health-behaviours should thus improve health and longevity.

During political separation, cardiovascular disease risk factors (e.g., smoking, obesity, physical inactivity, alcohol consumption) were more prevalent amongst Eastern Germans (particularly males) than their Western counterparts (Luy, 2004). However, most of these factors started to converge in the decade following reunification (Prütz et al., 2014; Lampert et al., 2019).

Given that most improvements in East German life expectancy came from falling death rates from cardiovascular causes, it would be plausible to suggest that changing health behaviours of East Germans post-reunification were a likely cause of reduced health inequalities between East and West (Gjonça et al., 2000). However, available evidence to date provides little support for this explanation (Luy, 2004). Alternative explanations suggest that differences in *past* female smoking behaviours (with female smoking more prevalent in the West prior reunification) was one of the main contributors to the observed life expectancy convergence (Myrskylä and Scholz, 2013). However, overall, the evidence base on health behaviours remains small and inconclusive.

#### 1.2.3 Psychosocial pathway

The psychosocial explanation of health inequalities examines how social oppression can lead to negative feelings (e.g., subordination, inferiority, fear) and that these feelings can have biological impacts with long term consequences for health. For example, cohort studies have shown that chronic stress has an impact on the body, leading to physical and mental ill-health (Marmot and Wilkinson, 2005). Thus, we could expect that the changes to the East Germany political system – notably the increases in political freedom and democracy – to result in better health outcomes for East Germans post-reunification.

Similarly, operating via the ‘Psychosocial’ pathway, expansions in social security benefits (discussed above) may also impact health and wellbeing indirectly. For example, additional income provided by benefits may increase financial security and lower social status anxiety. Additionally, improved redistributive measures may help reduce the overall levels of income inequality which has been shown to be causally linked with numerous population health indicators, including mortality (Wilkinson and Pickett, 2010).

Prior to reunification, self-reported life satisfaction was lower in the East than West Germany (Frijters et al., 2004). Given the dramatic rises in unemployment and social stress immediately following reunification (Krueger and Pischke, 1992; Mielck et al., 2000), life satisfaction dropped even further in the East in the early 1990s (Frijters et al., 2004). Suicide rates, particularly among men rose in the transition period (1989–1991). However, since 1992 they decreased in the East converging to Western levels by the early 2000s (Lampert et al., 2019).

Life satisfaction, too, had improved following the initial drop, with positive changes primarily attributed to increased political freedom and to a lesser extent, improved material living conditions (Frijters et al., 2004). There is evidence to suggest that improved life satisfaction can translate into greater longevity (Hu et al., 2017), however, whether it has contributed to the reduced life expectancy gap following German reunification, remains unclear.

Overall, previous evidence on life expectancy trajectories post-reunification points to material factors – mainly consisting of improvements in healthcare technology – as main drivers of the substantial narrowing of the gap in longevity between East and West Germany, with little evidence on other potential material factors, such as social security. Similarly, evidence regarding behavioural and psychosocial factors is also very limited, suggesting that a more comprehensive approach to addressing the main contributors to the narrowing of the East *vs* West life expectancy is needed.

Based on previous theoretical and empirical literature, as well as available data, our study focuses on the following factors that could help explain the changes in East-West life expectancy gap: living conditions (social security benefits) and healthcare (‘Material’ pathway); alcohol-related health behaviours (‘Behavioural’ pathway); and life satisfaction (‘Psychosocial’ pathway).

## 2 Methods

### 2.1 Analytical sample

We used federal state level data for 15 regions in Germany (all regions except Berlin), with East and West including five and eleven states, respectively (where East includes: Mecklenburg-Vorpommern, Brandenburg, Saxony, Saxony-Anhalt, and Thuringia; West includes: Bavaria, Bremen, Hamburg, Hessen, Lower Saxony, North Rhine–Westphalia, Rhineland-Palatinate, Schleswig-Holstein, Baden-Württemberg, and Saarland. In line with previous literature (e.g., Myrskylä and Scholz, 2013), we excluded the state of Berlin due to its historical separation into East and West and the lack of data for the separate halves. Owing to data availability, the time frame of our analysis is 1994–2020.

### 2.2 Outcome variables

The main outcome of interest in our analysis is life expectancy at birth for males and females, expressed in months. We obtained regional life expectancy estimates from the Federal Institute for Population Research (Mühlichen, 2023; Das Bundesinstitut für Bevölkerungsforschung, 2024). For further analyses, we also used estimates for remaining life expectancy at age 65, obtained from the same source.

### 2.3 Explanatory variables

The variables used to explore the ‘material pathway’ include social security benefit expenditure per capita and, for healthcare, given data availability for our analysis timeframe, the number of angiography workstations per 1000 residents in each region. (Data for variables such as healthcare expenditure were not available by region and for most of the study time frame). Nevertheless, as shown in Appendix A, over the period 1994–2020, expenditure in diagnostic imaging, which includes angiography workstations, increased by 2.41 times, closely mirroring the 2.56-fold increase in total healthcare expenditure (Federal Statistical Office, 2024), thus making it a reliable proxy for total healthcare expenditure. Additionally, broader literature supports using healthcare technology metrics as evidence of indicators of healthcare investment and spending (Chandra and Skinner, 2012).

The data on social security benefits were sourced from the federal and state statistical offices (Statistische Ämter des Bundes und der Länder, 2024); the data on angiography workstations were obtained from the federal government’s online health reporting database (Die Online-Datenbank der Gesundheitsberichterstattung, 2024). All monetary values of the social security benefits (which include pensions, unemployment benefits and child benefits) are in euros and adjusted to 2015 price level. For further exploratory analyses, we also included the above components of social security as separate variables. These variables were sourced from the German Socio-economic Panel (GSOEP).

The GSOEP is a nationally representative panel of around 13,500 individuals living in 7000 households since 1984. In 1990, the panel was extended to include residents of the East of Germany (Goebel et al., 2019). For our analysis, we aggregated the individual social security benefit measures into regional averages for each year. The averages were weighted using individual survey weights to ensure sample representativeness (SEOP Group, 2022).

To explore the ‘Behavioural’ pathway, we investigated the effects of alcohol consumption in each region, proxied by alcohol-related traffic injuries and deaths per capita – the only variable with enough data available for the analysis period to investigate this pathway, sourced from the federal government’s online health reporting database (Die Online-Datenbank der Gesundheitsberichterstattung, 2024). Given that the variable was only available in certain years (in 1991; 1995; 2000; 2005; 2010; 2015–2021), we estimated a linear trend based on available data points to obtain estimates for the missing years. To investigate the impact of this assumption, as a sensitivity analysis, we also estimated models using only the original data.

Self-reported life satisfaction was used to explore the impact of the ‘Psychosocial’ pathway. The source for this variable (aggregated across regions) was the GSOEP.

Finally, it should be noted that GSOEP also includes data on self-reported alcohol consumption and smoking, but these variables were not included due to the limited number of years for which they were available (i.e., smoking is only available from 1998 and at irregular intervals; alcohol consumption is only available for years 2006, 2008, and 2010).

Given that we are interested in relative contributions of each variable, all explanatory variables were standardised relative to their means. Thus, they should be interpreted as one standard deviation change in explanatory variable ‘x’ is associated with ‘y’ change in months in life expectancy. For easier interpretation, where appropriate, we also provided results with changes in key independent variables expressed in percentages.

### 2.4 Other covariates

To account for socio-demographic changes within each region, such as migration, we also controlled for the following factors: population size, age composition (% working age population), and education (% with above high school education). Finally, to account for time trends that affect all regions (e.g., influenza outbreaks), we also included year dummies. The data for population size came from the federal and state statistical offices (Statistische Ämter des Bundes und der Länder, 2024); the data for the remaining variables were sourced from GSOEP (Goebel et al., 2019; SOEP, 2023).

### 2.5 Statistical analysis

As a first step, we plotted descriptive trends over time for each variable of interest. Next, we used pooled Ordinary Least Squares (OLS) regression and fixed effects linear regression models to estimate the relationship between our key exposure variables and life expectancy. The pooled OLS model (Model 1), adjusted only for year, was used to examine the crude association between changes in main exposure variables and life expectancy, without accounting for region-level confounding.

We then explored how within-region changes in explanatory variables are associated with within-region changes in life expectancy using fixed effects models (Model 2).

Fixed effects account for the influence of fixed regional differences on health, such as geography and culture, by exploiting within-region variation only, and, as such, makes a more robust and internally valid analysis method than considering all states as two cross-sectional units (i.e., by comparing East and West Germany only) (Allison, 2009). To also minimise the potential sources of time-varying confounding, we included controls for regional-sociodemographic characteristics, including population size, education, proportion of working-age population (Model 3 – preferred specification).

To investigate the differential impact of the main explanatory variables on East vs. West and thus the extent to which each factor has contributed to the narrowing gap, we interacted the indicator for East (vs. West) with each of the main explanatory variables (Hu et al., 2017).

More specifically, for our main specification (Model 3), we estimated models of the following form:

$$LE_{it}=\beta_{1}F_{it}*East+\beta_{2}X_{it}+\alpha_{i}+\varepsilon_{it}$$

where *i* denotes region; *t* denotes calendar year (1994–2020).

In this equation, *LE* is life expectancy in years (at birth and at 65 years); *F*_*it*_ is a vector for the exposure factors of interest (i.e., social security benefits, number of angiography stations, alcohol-related traffic injuries and deaths, and life satisfaction); *X*_*it*_ is a vector of control variables, including population size, education, proportion of working age population; *α*_*i*_ is a region fixed effect, and *ε*_*it*_ is a random error term. All models were estimated for males and females separately and included dummies for each year. To account for potential autocorrelation and heteroskedasticity, we used robust standard errors, clustered at regional level. All models were estimated using Stata v.18 (StataCorp, 2023).

To better understand the contribution of different components to social security benefits (i.e., child, unemployment, and retirement benefits), in an additional analysis we included these three components instead of the overall social security benefit variable.

### 2.6 Robustness checks

We have investigated the validity of our results using several robustness checks, as follows.

- To investigate the impact of excluding Berlin, we estimated alternative models with Berlin as part of either East or West Germany;
- To investigate the validity of the linear trend assumption for deriving the alcohol-related traffic injuries and deaths variable, we estimated models with the original variable (which contained missing values for certain years);
- To investigate the validity of the linearity assumption of all exposure variables, we estimated models with logarithmic values;
- To investigate the suitability of angiography stations as proxy for healthcare availability, we instead used number of MRI machines;
- Finally, to investigate if potential lag exists behind exposure-outcome associations, we estimated alternative models with and one- and two-year lags of exposure variables.

## 3 Results

The total number of observations potentially available for the analysis was 405 (15 regions x 27 years). However, six observations had to be excluded due to missing data for the Western region Saarrland in GSOEP between 1994 and 2000, resulting in 399 observations.

### 3.1 Descriptive trends

**Fig. 1:**
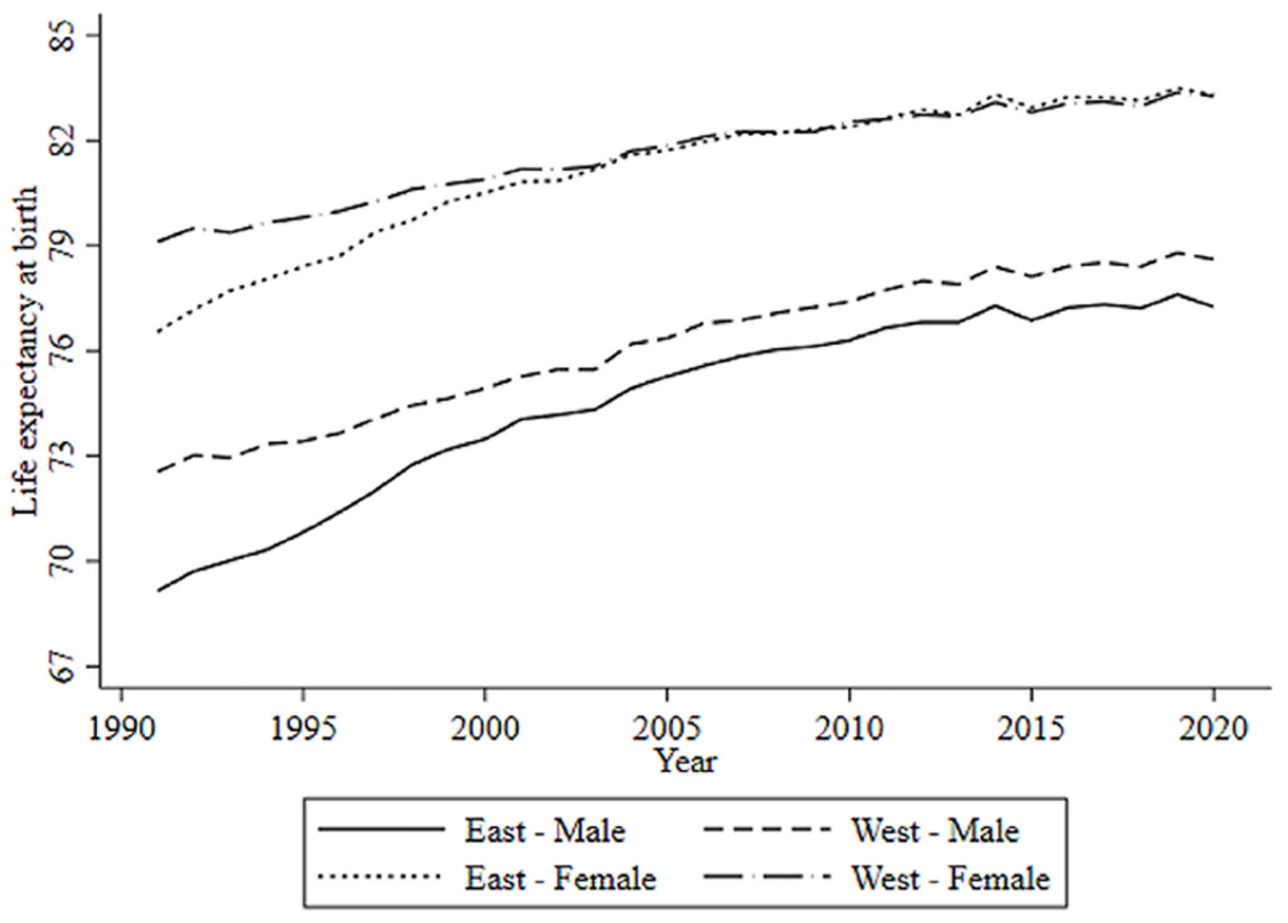
Life expectancy at birth in East and West Germany, by sex.

**Fig. 2:**
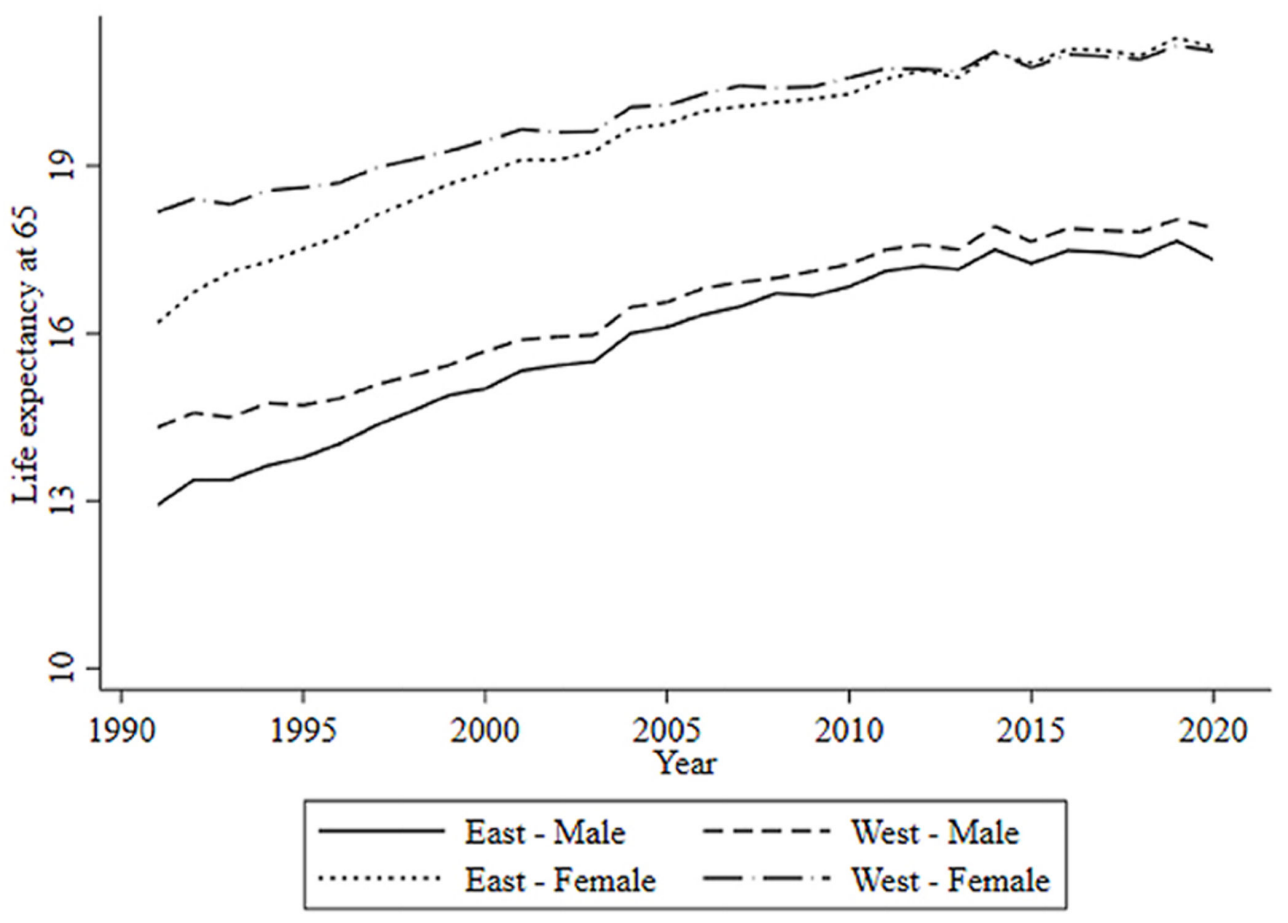
Remaining life expectancy at age 65 in East and West Germany, by sex.

Figs. 1 and 2 illustrate the trends in life expectancy at birth and remaining life expectancy at age 65 respectively, for East and West Germany. For females, life expectancy at birth converges in around 2010 and remaining life expectancy at age 65 converges later – in around 2015. For males, there is a narrowing in life expectancy gap at birth: from 3.5 years in 1990 to 1.5 years in 2020 but the trends in East and West never fully converge. Similar trends can be seen for remaining life expectancy at age 65.

**Fig. 3:**
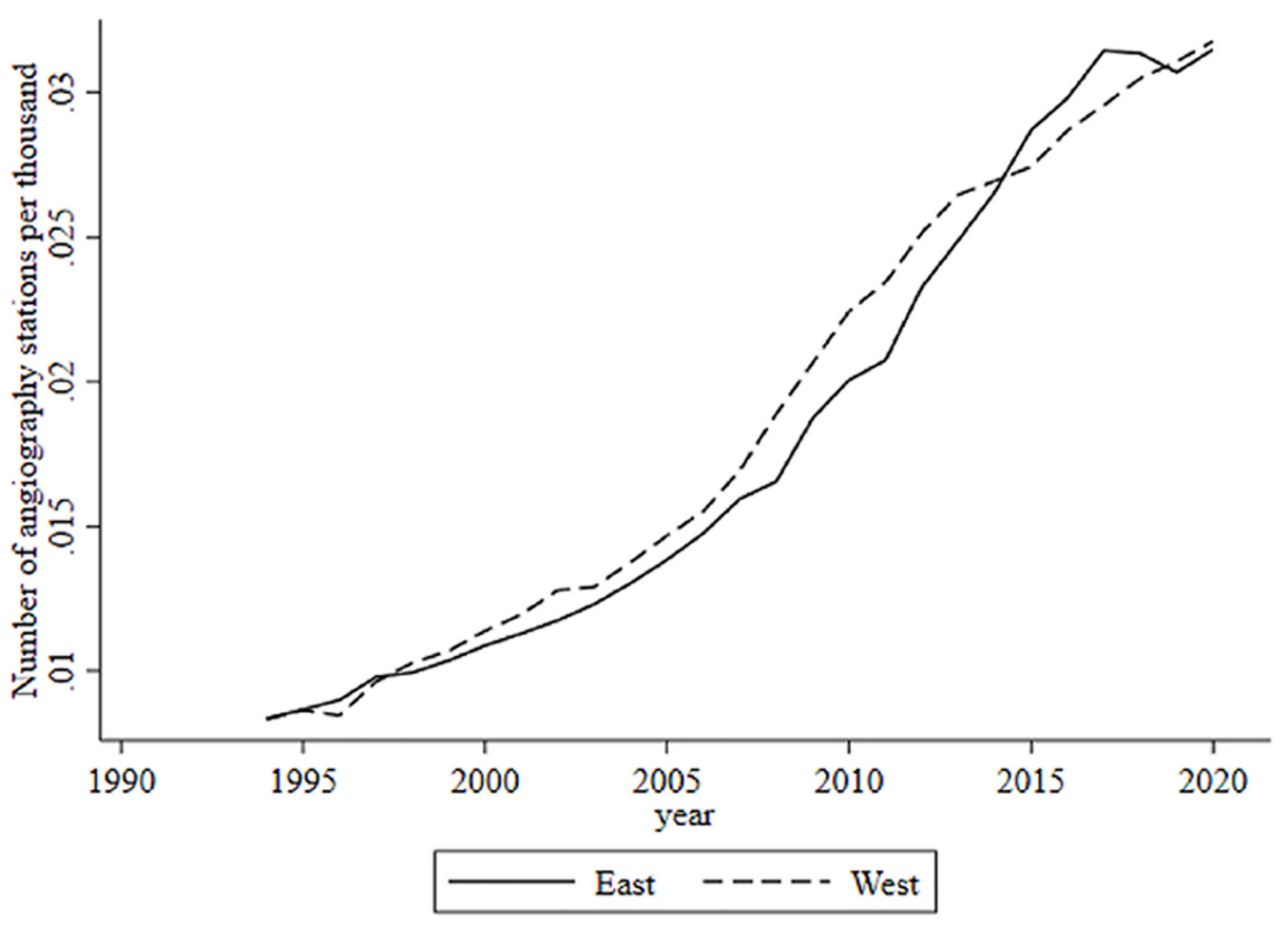
Number of angiography workstations per 1000 population in East and West Germany.

Fig. 3 shows the trends in the number of angiography workstations per 1000 of population for East and West Germany. We can see a clear upward trend in both East and West. However, in around 2015, East overtook the West.

**Fig. 4:**
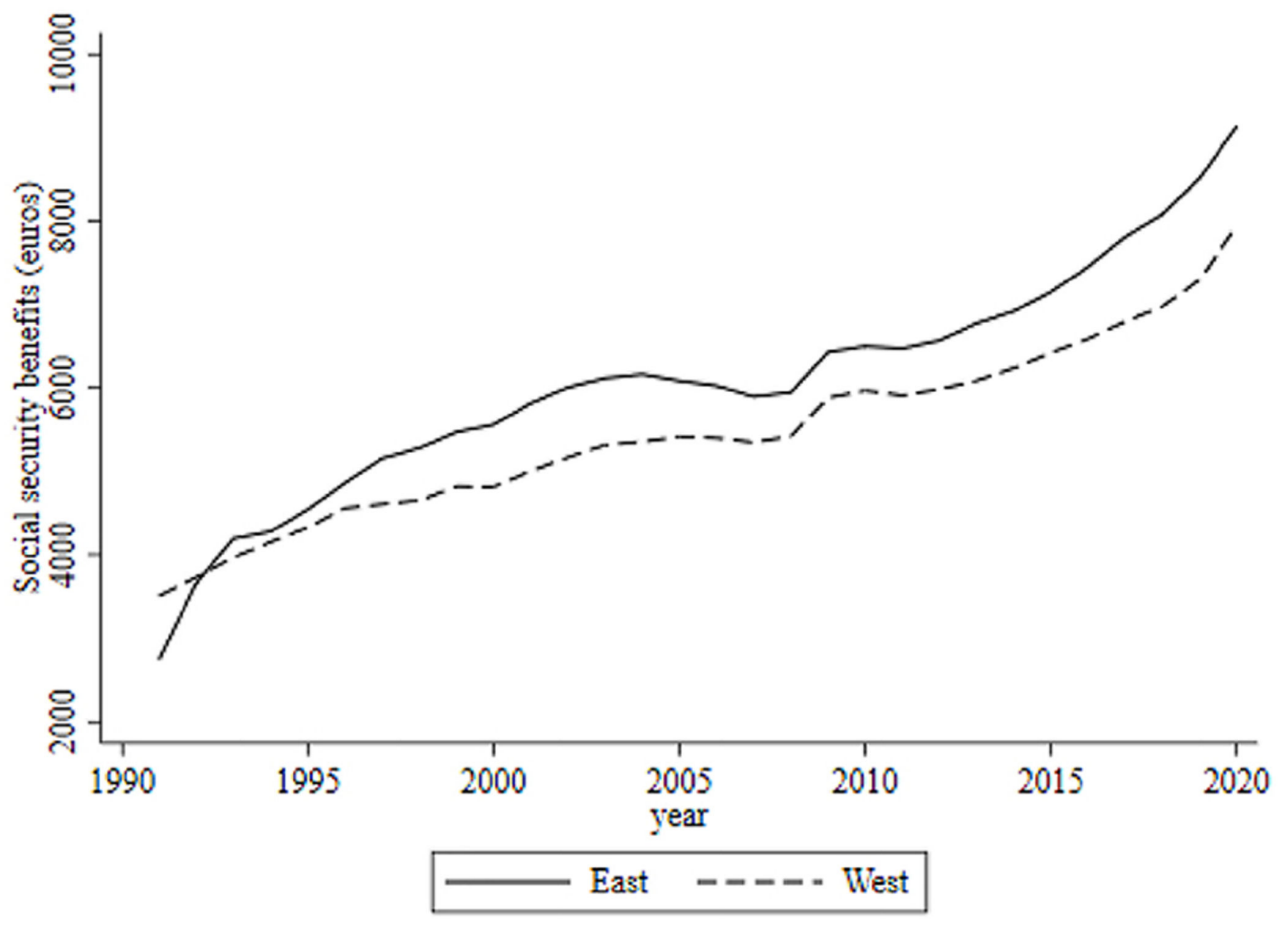
Social security benefits per capita per annum in East and West Germany.

In Fig. 4, we can see a rapid convergence of social security benefits per capita between East and West following reunification, with social security benefits in the East overtaking those in the West before 1995.

**Fig. 5:**
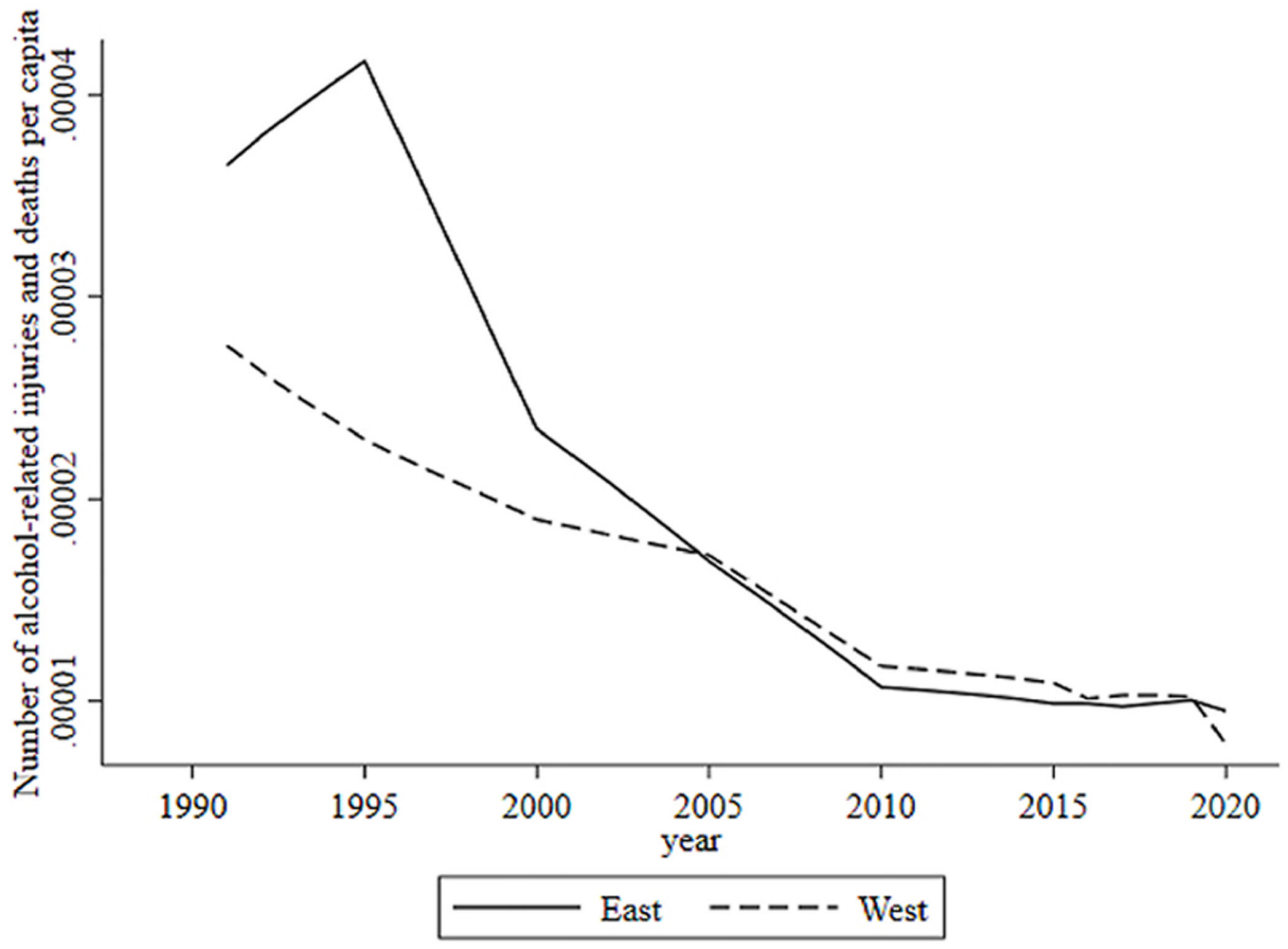
Number of alcohol-related traffic injuries and deaths per capita in East and West Germany.

In Fig. 5, we can see trends in the number of alcohol-related traffic injuries and deaths per capita in East and West Germany. Consistent with previous literature (Pechholdová et al., 2017), there is a large spike in alcohol-related injuries and deaths in mid-1990s in the East, reflecting the immediate post-transition crisis. However, the rates gradually come down and converge with Western levels in early 2000s.

**Fig. 6:**
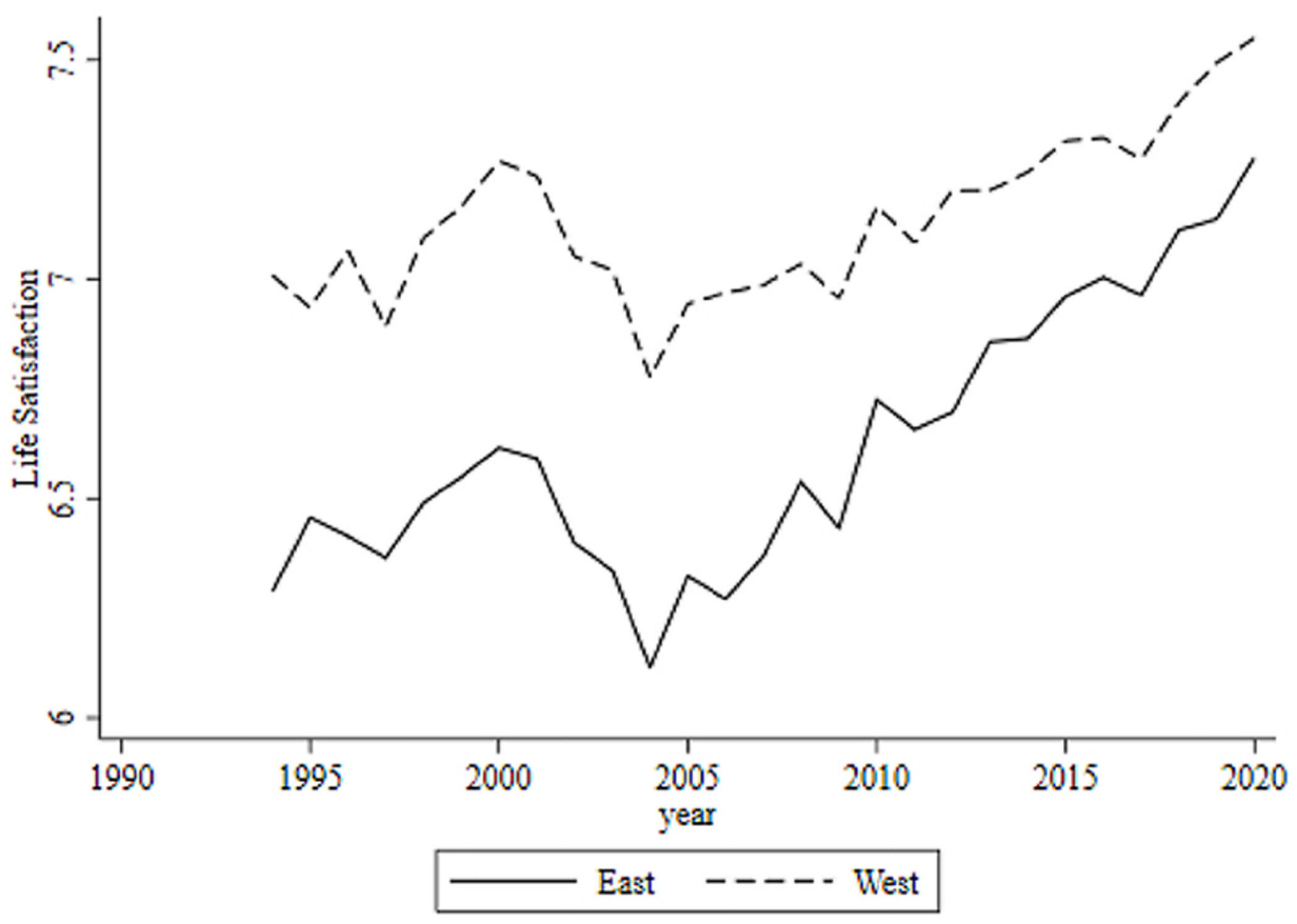
Average life satisfaction in East and West Germany.

Finally, Fig. 6 illustrates trends in life satisfaction between East and West Germany. Between 1990 and 2020 both East and West Germans have experienced improvements in life satisfaction, however, improvements were faster in the East, resulting in a much narrower gap in life satisfaction by 2020.

### 3.2 Statistical analysis results

Results for life expectancy at birth for males and females, are illustrated in Table 1. We find no association between any of our exposure variables and life expectancy at birth for West Germany (except for a positive association between higher social security benefits and life expectancy for men (p < 0.1), with every standard deviation increase in social security benefits leading to 5.5 [−0.78,11.72] additional months in life expectancy).

Results for East Germany, on the other, hand suggest that there is a positive association between increases in social security benefits and life expectancy for both males and females, with greater associations for males. Results in the fully adjusted model (Model 3) indicate that for every standard deviation increase in social security benefits per capita, life expectancy increases by 1.6 [0.001,0.26] months for women and by 2.4 [0.088,0.311] months for men, relative to their counterparts in West Germany. For ease of interpretation (in percentages), we have also included the social security benefits variable in log form. The results suggest that for every 10% increase in social security benefits, life expectancy increases by 1.05 [0.68; 1.41] months for men, by 0.57 [0.18; 0.97] months for women.

There is a negative association (p < 0.1) between increased number of angiography workstations and life expectancy at birth for males with coefficient equal to −2.1 [−4.53,0.33]. Alcohol-related traffic injuries and deaths, too, have a negative relationship with life expectancy at birth in East vs. West Germany, whereas for life satisfaction there is no association.

We observe similar overall patterns for life expectancy at age 65 (see Table 2), however several differences should be noted. First, there is a small positive relationship between the number of angiography stations and male life expectancy in West Germany (while no relationship for either males or females in the East). Second, there is a positive relationship between life expectancy between alcohol-related traffic injuries and deaths for West Germany, with coefficient equal to 3.2 [−0.17,6.60]. Finally, looking at interaction coefficients, the positive association between life expectancy and social security benefits only holds for women, with coefficient equal to 1.6 [0.20,3.04]. When expressed in percentage terms, the results relating to the social security variable suggest that a 10% increase in benefits is associated with an increase of 0.38 [0.06; 0.70] months in life expectancy at 65 for women.

Further analysis in Appendix B suggests that social-security related improvements in life expectancy both at birth and at 65 years were driven primarily by increases in pension benefits.

### 3.3 Results of robustness checks

The results of these analyses are illustrated in Appendices C-G,suggesting that the overall conclusions remain unchanged. The robustness of our models to alternative specifications is reassuring and provides support for the internal validity of our findings.

## 4 Discussion

### 4.1 Summary and contextualisation of main findings

Within two decades following reunification, East Germans had experienced significant increases in life expectancy relative to West Germans which meant that the life expectancy gap narrowed markedly for men and disappeared for women within less than two decades. This is a remarkable achievement particularly in the wider context of post-socialist transition economies, many of which had experienced an unprecedented mortality crisis following the collapse of socialism in the 1990s (Stuckler et al., 2009).

Unlike in other post-socialist economies, however, the marked rise in life expectancy since reunification was accompanied by soaring public spending and social infrastructure designed to eliminate regional disparities between East and West. Our aim was to quantify the potential factors contributing to the narrowing life expectancy gap between the two halves of Germany.

Drawing on the post-Communist literature and informed by theories of health inequalities and the political economy of health, we have identified four factors as potential contributors: social security benefit expenditure and number of angiography workstations (‘Material pathway’), alcohol-related traffic injuries and deaths (‘Behavioural pathway’), and life satisfaction (‘Psychosocial pathway’).

We have found that increasing social security benefit expenditure was the only factor contributing to narrowing inequalities in life expectancy at birth for both men and women as well as a contributor for narrowing life expectancy gap at age 65, but only for women – a finding consistent with the fact that older females were amongst the greatest (financial) beneficiaries of reunification due to their long working histories relative to their West German counterparts (Vogt and Kluge, 2015). Relatedly, previous evidence suggests that older individuals (aged 60+) benefitted the most from reunification in terms of life expectancy gains (Grigoriev and Pechholdová, 2017). While this has been mainly explained by improvements in healthcare (and the resulting lowering mortality from primarily cardiovascular causes) (Vogt and Vaupel, 2015), our study indicates that expansions social security benefits, and in particular, pensions, may have also contributed.

The remaining factors (angiography workstations, alcohol-related traffic injuries and deaths, and life satisfaction) had either null or negative associations and were thus unlikely to contribute to the convergence in life expectancy.

Our findings provide support for the materialist explanation of health inequalities, adding to the considerable evidence base on the importance of material factors for health (Moor et al., 2017). Given the political nature of reunification, our results also highlight the importance of political decisions and public policies in shaping health inequalities – a finding in line with political economy of health approach.

In particular, our results suggest that the significant monetary transfers to East Germany following reunification have likely positively contributed to their rapid improvement in life expectancy, markedly reducing the regional health inequalities between East and West. West Germany’s well-established and comparatively generous social security system was implemented in the East and used to provide a safety net to East Germans during the turbulent economic changes (Hall and Ludwig, 1994). As a result, pensions and unemployment benefit levels improved leading to a fall in poverty (Vaizey, 2016). Relative household poverty (measured as income less than 60% of the median) levels fell in the East post-reunification from 25% in 1991 to 16% in 2000 (Federal Government Commissioner for the New Federal States, 2018). In the same period, it rose from 10% to 13% in the West (ibid).

Forty years on from reunification, whilst there are still considerable economic gaps with the West (e.g., the states of the former East Germany are still poorer and experience higher levels of unemployment (van Raalte et al., 2020)), the East is doing relatively well by European standards – several states in Eastern Germany have higher GDPs than other regions in Western Europe (such as the North of England) and the per capita GDP in Eastern Germany, overall, is higher than that of countries such as Portugal (Rehnberg, 2020; Eurostat, 2023).

Our findings are consistent with broader literature on the importance of social transfers, poverty reduction and health inequalities. For example, an international comparative study of eighteen OECD countries by Lundberg et al. (2008) has found that more generous family and pension policies can lead to lower infant mortality and old-age excess mortality respectively. Similarly, recent evidence from the UK suggests that cuts in local government funding have been associated with widening disparities in regional mortality (Alexiou et al., 2021). Social security has also been identified as a key ‘leveller’ of health inequalities in countries as diverse as Brazil and the USA (Bambra, 2022).

Our results are also consistent with findings from international systematic reviews on welfare transfers and social security. For example, a systematic review by Simpson et al. (2021) has found that expansions in social security are associated with lowering mental health inequalities, whereas contractions have the opposite effect. Likewise, an umbrella review of macroeconomic factors and health inequalities has similarly found that more generous welfare states lead to better population health outcomes and lower inequalities (Naik et al., 2019).

However, suggesting null or weak negative associations between increases in healthcare availability and life expectancy, our findings are not entirely in agreement with literature on German reunification. Previous literature focusing on reunification and mortality generally finds advances in medical care as the main positive factor contributing to narrowing life expectancy gap (Nolte et al., 2002; Vogt and Kluge, 2015; Vogt and Vaupel, 2015).

The ambiguity of the role of medical services in alleviating mortality in economically developed societies is also reflected in the cross-country empirical literature. A recent systematic review by Scheiring et al. (2019), for example, has found little support for healthcare expenditure in preventing mortality post-socialist mortality crisis, with five out of eight included studies showing no association. These findings are in line with broader European-level evidence which has identified spending on welfare (as opposed to healthcare) as the key predictor of mortality differences across 15 countries (Stuckler et al., 2010). The discrepancies in these findings likely reflect different methodologies, variable definitions, contexts and time frames studied, and indicate that further research, particularly using individual level data, is still needed to better understand the role of healthcare and social security in explaining health inequalities both at regional and international levels.

Our results have clear policy implications for other countries. Germany took a long-term approach to improving living conditions – and health – in the East. These improvements were made possible by the deep and sustained cross-party political commitment to reunify Germany as fully as possible: £70 billion a year for 25 years was invested in ‘levelling up’ East Germany, funded by a special solidarity surcharge (Centre for Cities, 2021). There are lessons for other countries seeking to reduce regional inequalities here. For example, in post-Brexit UK, regional imbalances have become increasingly politically salient, and both main political parties have committed to ‘levelling up’ health across the regions and countries of the UK by reducing regional economic, social and healthcare inequalities. However, the funds so far committed (£4.8 billion over four years) – and the policies implemented – to date are well below the German model that we have outlined here (ibid). Our findings provide support to the notion that, to reduce entrenched health inequalities, sustained and multi-faceted action across different sectors of the economy is needed. This may include economic investment and renewal, improvements in health prevention, promotion and treatment; industrial strategy; as well as social policies.

### 4.2 Strengths and limitations

Our study has several strengths. First, we used regional level data covering 27 years, thus enabling us to study life expectancy convergence over a longer time frame than most of the previous studies (largely focusing on the decade immediately post reunification). Secondly, having panel data has allowed us to focus on within region variation, thus eliminating the role of time invariant differences between regions, strengthening the internal validity of our findings. Furthermore, to minimise the effects of migration on our results, we included controls for the proportion of working age population as well as for education level. Additionally, we investigated life expectancy at 65 as an outcome which should be relatively unaffected by migration, given that it was the younger population that was more likely to leave East Germany following reunification (Burda and Hunt, 2001).

However, our study is not without limitations. For example, we were unable to investigate the period before or immediately after reunification, thus likely missing an important period for convergence in life expectancy. Relatedly, data limitations meant that we had limited choice for operationalising the main variables of interest which may help explain some of the less intuitive results. For example, the negative coefficient for angiography workstations for men might be indicative of the crudeness of this variable as a proxy of healthcare availability. However, to the best of our knowledge, this was amongst the only variables from official sources available at regional level to measure healthcare availability following reunification (e.g., healthcare expenditure data is not available by region dates back only to 2008). Additionally, sensitivity analyses using MRI machines as an alternative measure provided very similar conclusions.

Another important limitation of aggregate-level data analysis is that we could not investigate the specific mechanisms between higher social security expenditure and life expectancy. Such mechanisms could include, for example, increased consumption of healthy goods and services, better housing and overall living standards, resulting from the increases in income. Alternatively, social security benefits may improve overall health by improving psychosocial wellbeing (e.g., by lowering financial insecurity). Analyses of individual-level mechanisms linking welfare transfers and mortality might be possible in the near future, however, as the use of digital trace data in the social sciences accelerates and new linkages between administrative and survey data become possible (Keenan et al., 2022), highlighting this as a potential avenue for future research.

Relatedly, our analysis was not able to unpick the apparent differences in the evolution of male and female life expectancy gaps following reunification. Namely, while the gap between East and West had disappeared for women in little over a decade following reunification, a persistent gap (of around 1 year) remains for men. This gap is largely driven by higher rates of cancer and cardiovascular disease in middle-aged men in the East (aged 30–59 years) (Grigoriev and Pechholdová, 2017). Prior research suggests that compositional differences in individual income between East and West is a likely contributing factor driving the mortality disadvantage in the East (Grigoriev et al., 2019). Indeed, low-income is an established risk factor both for cancer and cardiovascular disease (Larsen et al., 2020; Minhas et al., 2023). Another important factor that has likely contributed to the persisting male life expectancy difference is the long-lasting negative effects of the immediate post-reunification shock, whereby high levels of unemployment due to rapid deindustrialisation had a significant negative impact on young men in terms of high alcohol consumption and stress (Grigoriev and Pechholdová, 2017; Lampert et al., 2019). However, overall, the evidence regarding the persistent East-West disparity in male life expectancy remains inconclusive and warrants future research at more granular level to help elucidate the underlying causes of death and their drivers.

As with all population-based analyses, our results are susceptible to ecological fallacy. We also cannot rule out the possibility that the associations observed were caused by other confounding factors for which we were not able to control. However, we attempted to overcome this limitation by focusing on within region variation and controlling for national time trends and regional socio-demographic factors.

Finally, it should be noted that, given a complex interplay of these factors, the relative contributions of each factor are likely to be influenced by other variables, making it difficult to completely isolate single factors (Grigoriev and Pechholdová, 2017). However, our analysis should still give us an indication of the relative importance of each factor, given that we control for other important characteristics and factors.

## 5 Conclusion

Based on regional data for 15 states of Germany between 1994 and 2020, our study suggests that social security expenditure was the most important factor explaining narrowing health inequalities between East and West Germany during this period, as opposed to behavioural or psychosocial factors. This provides additional support for the relevance of the materialist hypothesis and the political economy of root causes of health inequalities and suggests that increasing social security expenditure could be an effective policy-based tool for closing health gaps across regions with different levels of economic development.

## Supplementary Material

**Appendix A.Supplementary data**

Supplementary data to this article can be found online at https://doi.org/10.1016/j.socscimed.2024.117436.

## Figures and Tables

**Table 1:** Associations between male and female life expectancy at birth and hypothesised contributing factors including interactions for East vs. West Germany.

	Model 1^a^			Model 2			Model 3	
	Women	Men		Women	Men		Women	Men
**Ref. West:**								
Angiography workstations	−0.171 [-1.429,1.087]	0.105 [-0.994,1.205]		0.318 [-0.704,1.340]	0.579 [-0.215,1.374]		−0.131 [-0.971,0.708]	0.085 [-0.534,0.704]
Social security benefits	2.432 [-4.218,9.083]	2.763 [-9.041,14.567]		2.568 [-4.944,10.08]	5.298 [-2.092,12.688]		3.025 [-2.990,9.041]	5.481* [-0.767,11.729]
Alcohol-related injuries and deaths	−0.575 [-3.285,2.135]	−1.587 [-7.668,4.493]		0.017 [-1.663,1.698]	1.051 [-1.195,3.297]		0.602 [-0.935,2.140]	1.364 [-1.072,3.799]
Life satisfaction	0.539 [-0.386,1.465]	0.491 [-0.216,1.198]		0.395 [-0.332,1.121]	0.216 [-0.355,0.788]		0.257 [-0.444,0.958]	−0.022 [-0.515,0.471]
East	−3.821 [-9.883,2.242]	−16.730*** [-27.752,-5.708]		NA	NA		NA	NA
**Additional effects for East:**								
East X Angiography workstations	0.218 [-1.824,2.260]	−2.344 [-6.008,1.320]		−0.732 [-2.740,1.275]	−2.993** [-5.850,-0.135]		−0.000 [-1.626,1.625]	−2.101* [-4.533,0.331]
East X Social security benefits	2.209*** [1.075,3.343]	3.263*** [1.422,5.104]		1.244 [-0.676,3.163]	1.720** [0.280,3.159]		1.564** [0.013,3.115]	2.393*** [1.057,3.729]
East X Alcohol-related traffic injuries and deaths	−6.527*** [-8.104,-4.951]	−6.857*** [-10.782,-2.932]		−6.447*** [-7.617,-5.277]	−7.813*** [-10.819,-4.807]		−7.072*** [-8.142,-6.001]	−8.752*** [-11.613,-5.891]
East X Life satisfaction	−0.694** [-1.305,-0.083]	−1.040 [-2.542,0.462]		−0.471 [-1.250,0.309]	−0.756 [-2.343,0.830]		−0.403 [-1.151,0.345]	−0.591 [-1.788,0.605]
Observations	399	399		399	399		399	399

95% confidence intervals in brackets

* p < 0.1,

** p < 0.05,

*** p < 0.001.

a Model 1: Pooled OLS with year controls; Model 2: FE with year controls; Model 3: FE with year + socio-demographic controls.

**Table 2:** Associations between male and female life expectancy at 65 years and hypothesised contributing factors including interactions for East vs. West Germany.

	Model 1^a^			Model 2			Model 3	
	Women	Men		Women	Men		Women	Men
**Ref. West**:								
Angiography workstations	−0.438 [-1.775,0.900]	−0.690 [-2.013,0.634]		−0.040 [-0.979,0.898]	−0.260 [-1.249,0.729]		−0.271 [-1.057,0.515]	−0.462 [-1.383,0.459]
Social security benefits	2.880 [-1.551,7.310]	3.768 [-1.014,8.550]		1.324 [-2.633,5.281]	3.175* [-0.296,6.646]		1.400 [-1.980,4.781]	3.214* [-0.170,6.598]
Alcohol-related injuries and deaths	0.774 [-1.174,2.721]	0.924 [-1.180,3.027]		0.191 [-1.425,1.807]	1.082* [-0.057,2.222]		0.352 [-1.273,1.978]	1.188** [0.175,2.202]
Life satisfaction	0.260 [-0.716,1.235]	0.140 [-0.718,0.997]		0.203 [-0.538,0.945]	0.031 [-0.503,0.564]		0.144 [-0.592,0.879]	-0.044 [-0.567,0.479]
East	−5.542*** [-8.751,-2.334]	−7.252*** [-11.561,-2.943]		NA	NA		NA	NA
**Additional effects for East:**								
East X Angiography workstations	1.484* [-0.206,3.173]	0.306 [-1.425,2.037]		0.556 [-0.666,1.778]	−0.603 [-1.644,0.438]		0.881 [-0.339,2.101]	−0.285 [-1.244,0.675]
East X Social security benefits	1.847*** [0.681,3.014]	0.469 [-1.403,2.341]		1.445** [0.060,2.829]	−0.126 [-2.075,1.823]		1.617** [0.195,3.039]	0.095 [-1.955,2.144]
East X Alcohol-related traffic injuries and deaths	−3.899*** [-5.326,-2.472]	−3.639*** [-5.241,-2.038]		−3.297*** [-4.515,-2.080]	−3.325*** [-4.711,-1.939]		−3.516*** [-4.706,-2.327]	−3.601*** [-5.031,-2.171]
East X Life satisfaction	−0.077 [-0.607,0.453]	−0.333 [-0.851,0.184]		0.072 [-0.498,0.643]	−0.173 [-0.771,0.425]		0.105 [-0.477,0.687]	−0.123 [-0.582,0.336]
Observations	399	399		399	399		399	399

95% confidence intervals in brackets.

* p < 0.1,

** p < 0.05,

*** p < 0.001.

a Model 1: Pooled OLS with year controls; Model 2: FE with year controls; Model 3: FE with year + socio-demographic controls.

## Data Availability

Most data used in our analyses is publicly available. We do not have permission, however, to share the original GSOEP dataset and the regional life expectancy at 65 data.
